# Increased interleukin-18 level contributes to the development and severity of ischemic stroke

**DOI:** 10.18632/aging.102253

**Published:** 2019-09-16

**Authors:** Yong Hao, Jie Ding, Ronghua Hong, Shuwei Bai, Ze Wang, Chengjun Mo, Qiang Hu, Zezhi Li, Yangtai Guan

**Affiliations:** 1 Department of Neurology, Renji Hospital, Shanghai Jiao Tong University School of Medicine, Shanghai, China; 2 Department of Psychology, Qiqihar Mental Health Center, Qiqihar, China

**Keywords:** Interleukin-18, ischemic stroke, association, meta-analysis, cross-sectional study

## Abstract

Although interleukin-18 (IL-18) has been implicated in the pathophysiology of stroke, research findings concerning IL-18 level in stroke have been inconsistent. Thus, we performed a cross-sectional study in patients with first-episode ischemic stroke and then extracted relevant data from databases to validate our results. A total of 252 patients and 259 healthy subjects were recruited, and serum IL-18 level was evaluated in a cross-sectional study. Then, we extracted data and conducted a meta-analysis, including 2,928 patients and 3,739 controls to support our results. A 95% confidence interval for standardized mean difference (SMD) was calculated using a *Z* test. We found IL-18 was higher in stroke patients than in controls (2.39 ± 0.25 vs. 2.25 ± 0.28, F=8.60, p=0.004) and was negatively associated with the NIHSS scale (*r* = -0.14, *p*=0.028). A subsequent meta-analysis confirmed that IL-18 level was higher in stroke patients than in controls (SMD = 2.14, 95% CI = 1.54 ∼ 2.73, *P*< 0.001). IL-18 level increased with the severity of the stroke (*p*< 0.01). These findings revealed increased IL-18 level contributed to the development and severity of ischemic stroke, suggesting the potential of this biomarker to become an important reference for the early monitoring of ischemic stroke.

## INTRODUCTION

A stroke is also known as a cerebrovascular accident or, colloquially, a brain attack; ischemic stroke refers to the loss of brain function resulting from a disturbance in the blood supply to the brain [1]. Ischemic stroke has been documented to be one of the leading causes of mortality, with an even higher rate of chronic disability, and there are an estimated 6 million fatal cases of stroke annually across the world [2, 3]. Approximately one-third of 62 million stroke survivors suffer from neurological disabilities, which impose a heavy burden on families and society [4, 5]. Ischemic stroke results from either cerebral hypoperfusion or blood vessel blockage in the form of arterial embolism or thrombosis [6]. Currently, it is well established that old age, high blood pressure, high cholesterol, atrial fibrillation, diabetes history, cigarette smoking, and transient ischemic attacks are risk factors for stroke [7]. In addition to these multiple factors, convincing evidence has already implicated global brain inflammation and thrombo-inflammation as possibly critical factors affecting the evolution of pathology after a stroke and shaping stroke patients' long-term neurological outcomes [8–10]. Exploring the inflammatory mechanisms of ischemic stroke will undoubtedly promote rehabilitation, as illustrated by the observation that pretreatment with ACE inhibitors is a predictor of good outcomes owing to the pro-inflammatory effects of Ang II [11, 12].

IL-18, a pro-inflammatory cytokine, is encoded by the IL-18 gene in humans [13]. It is likely to be the first-line immune defense for the brain, primarily secreted by macrophage cells and mononuclear cells in humans [14]. It is initially expressed as the inactive precursor pro-IL-18 and is then converted into an active form by proteolytic cleavage, mainly by the cysteine protease caspase-1, which is also known as IL-1B converting enzyme (ICE) [15, 16]. Initially known as an interferon-gamma (interferon-γ)-inducing factor, IL-18 may enhance the Th1 immune response by stimulating the activation of natural killer (NK) and cytotoxic T cells [17, 18]. Previous studies have shown that IL-18 causes the polarization of T cells into T helper 1 (Th1) or 2 (Th2) cells, depending on the immunological context, and accelerates various adaptive and innate immune processes related to autoimmunity, inflammation, and infection [19, 20]. IL-18 level has been indicated to be associated with metabolic syndrome as well as type 2 diabetes mellitus [21, 22]. In addition, upregulated levels of IL-18 are believed to play a crucial role in ischemic stroke attacks [23]. In line with previous studies of the etiology of ischemic stroke, some researchers have proposed the hypothesis that a pro-inflammatory profile induced by increased IL-18 level creates a pro-thrombotic and pro-atherosclerotic environment, which may eventually contribute to stroke in older people [24]. Multiple lines of evidence have indicated that IL-18 level can predict the development of stroke [14, 25]; however, increased IL-18 level was not found in stroke patients in several other reports [24, 26]. The results were not consistent. The possible reasons for inconsistent results include differences in race, sample size, method, and illness duration in previous studies.

Thus, in the present study, we conducted a cross-sectional study in larger samples to investigate IL-18 level in acute first-episode stroke patients and to further examine the association of IL-18 level with the severity of stroke. In addition, we extracted previously published data from databases to validate the IL-18 level in stroke and investigate the main source of heterogeneity, resulting in inconsistent results among previous published studies.

## RESULTS

### Cross-sectional study

Demographic characteristics in patients and healthy controls were shown in Table 1. Age differed significantly between the patient group and control group; accordingly, age was included as a covariate in the following covariance (ANCOVA) model analysis. As serum IL-18 level was not normally distributed, we used a log transform to normalize the data distribution. We found that IL-18 level was significantly higher in patients than in healthy controls (2.39 ± 0.25 vs. 2.25 ± 0.28, F=8.60, p=0.004).

**Table 1:** Demographic characteristics in patients and healthy controls.

**Variable**	**Patients (N=252)**	**Controls (N=259)**	**Statistic**	***p***
Age (years, mean±SD)	66.4 ± 10.51	44.22 ± 12.51	t=21.78	<0.0001
Gender (male)	175 (69.80%)	165 (63.71%)	χ^2^=2.17	0.16
BMI	22.75±2.50	23.10±2.27	t=1.67	0. 10
Smoking	20 (7.94%)	14 (5.41%)	χ^2^=1.32	0.25
Duration of illness	32.01±4.54	-	-	-
NIHSS Scale	7.87±3.82	-	-	-
IL-18 level	2.39 ± 0.25	2.25 ± 0.28	F=8.60	0.004

Pearson correlation showed a significant association between IL-18 level and NIHSS scores (*r* = -0.16, *p*= 0.009). After adjusting for age, gender, BMI, smoking and disease duration, partial correlations were also found between IL-18 level and NIHSS scores (*r* = -0.14, *p*=0.028) (Supplementary Figure 1). Furthermore, we used multivariate analysis (stepwise) and found that both disease duration and severity were associated with IL-18 level in patients (B=0.02, *t*=4.52, *p*<0.001; B=-0.01, *t*=2.23, *p*=0.027, respectively), adjusting for age, gender, illness duration, BMI and smoking.

Partial correlation showed no association between IL-18 level and modified Rankin Scale (mRS) scores (*r*=0.02, *p*=0.77) or infarction volume (*r*=-0.02, *p*=0.80), adjusting for age, gender, BMI, smoking and duration of stroke. Furthermore, multivariable regression analysis showed no association between IL-18 level and mRS scores (B=0.11, *t*=0.30, *p*=0.77) or infarction volume (B=-2.45, *t*=0.26, *p*=0.80), adjusting for age, gender, illness duration, BMI and smoking. We found no significant association between mRS scores and IL-18 level (B=0.11, *t*=0.30, *p*=0.77).

We also analyzed the association between IL-18 level and other recognized ischemic stroke risk factors, including BMI, smoking, high blood pressure, diabetes, hyperlipidemia, atrial fibrillation and family history of stroke. Multivariate analysis (stepwise) also showed that BMI and diabetes were associated with IL-18 level (B=0.02, *t*=2.44, *p*=0.02; B=0.09, *t*=2.16, *p*=0.03, respectively), while smoking, high blood pressure, hyperlipidemia, atrial fibrillation and family history of stroke were not associated with IL-18 level (B=0.07, *t*=1.19, *p*=0.24; B=0.01, *t*=0.26, *p*=0.80; B=0.03, *t*=0.60, *p*=0.55; B=0.05, *t*=0.51, *p*=0.61; B=0.04, *t*=0.54, *p*=0.59; respectively).

We used the receiver operating characteristic (ROC) curve to calculate the cutoff value for illness severity. The cutoff IL-18 level between mild and moderate was 2.05 (log-transformed). The cutoff IL-18 level between mild and moderate was 2.63 (log-transformed).

### Summary of studies included for data extraction

**Figure 1:**
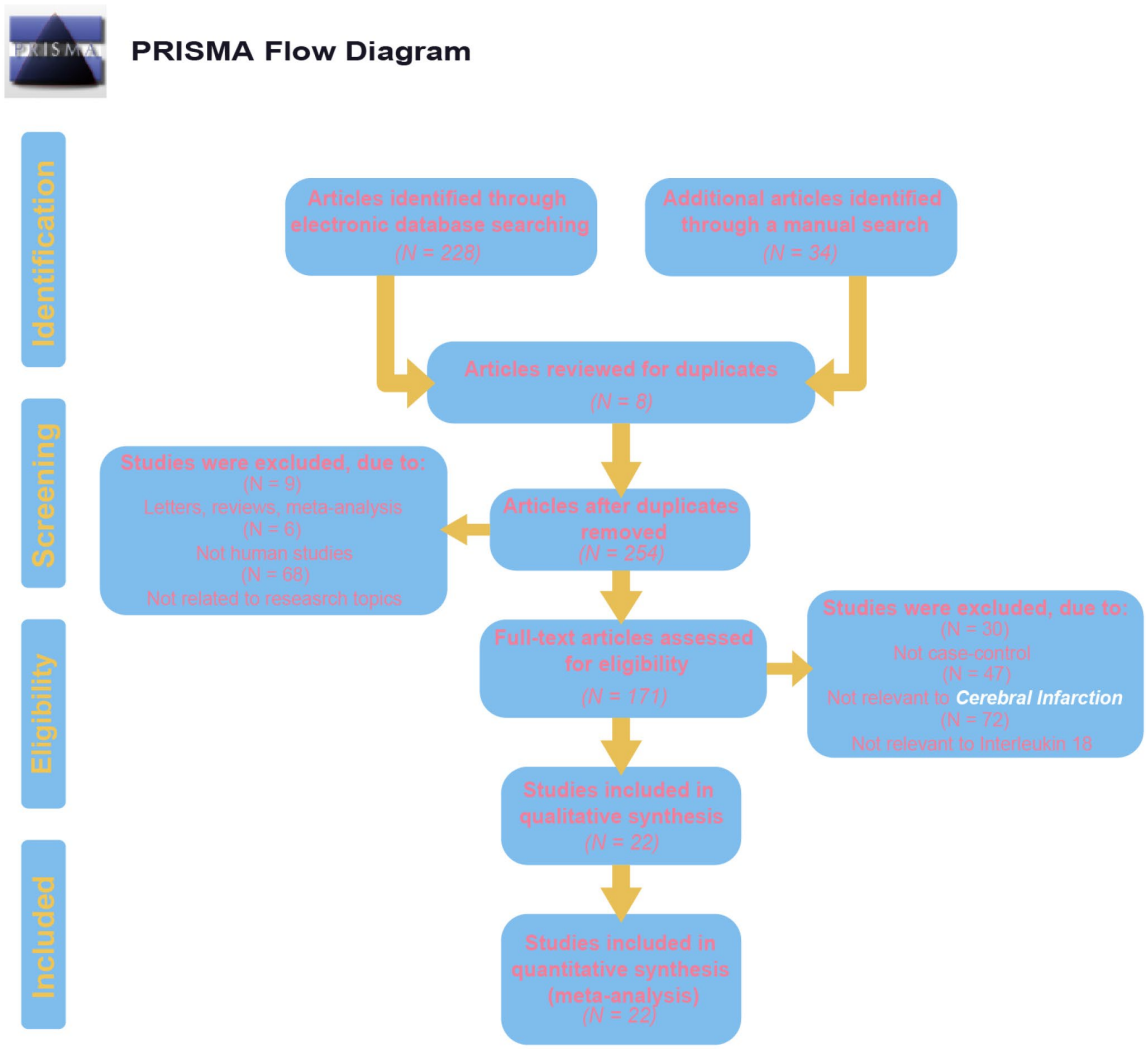
**Flow chart shows study selection procedure.** Twelve case-control studies were included in this meta-analysis.

**Figure 2:**
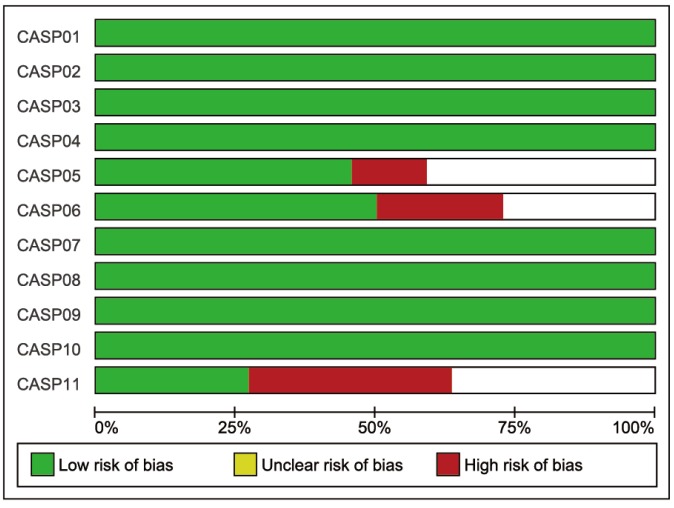
**The CASP score of the included literature.** The blue bar indicates lower risk of bias, and the red bar indicates higher risk of bias.

Initially, 262 eligible potential articles were selected. Then, duplicate studies were deleted, and the titles and abstracts of the remaining 171 studies were reviewed for relevance. After this review step, 149 articles were eliminated due to irrelevance, and 22 articles were ultimately selected to be incorporated into the meta-analysis, which included a total of 2,928 patients and 3,739 controls [14, 17, 24–44]. A flow diagram of the process for article selection and the reasons for exclusion is shown in Figure 1. Each of the included papers was of moderate to high quality. Among the 22 included studies, 15 were conducted in Asians, and the others were conducted in Caucasians. Regarding the methods used to measure IL-18 level, two studies were performed with Luminex xMAP, and the remaining 20 studies used ELISAs. The characteristics of studies and the IL-18 level at baseline in each study are shown in Table 2. The CASP scores of the included studies are shown in Figure 2.

**Table 2:** The baseline characteristics for included studies.

**Author**	**Year**	**Country**	**Ethnicity**	**Sample size**		**Gender (M/F)**		**Age (years)**		**Methods**
				**Case**	**Control**	**Case**	**Control**	**Case**	**Control**	
Zhao Y(2017)	2017	China	Asians	100	60	64/36	38/22	63.5±11.2	58.6±6.8	ELISA
Wang TR(2017)	2017	China	Asians	60	60	35/25	30/30	56.3±9.2	58.7±6.6	ELISA
Zou Q(2016)	2016	China	Asians	70	70	38/32	40/30	52.0±6.0	53.0±5.0	ELISA
Zhao HG(2016)	2016	China	Asians	112	55	61/51	31/24	62.0±10.0	60.0±7.1	ELISA
Lu YQ(2016)	2016	China	Asians	32	35	25/7	25/10	71.7±5.9	67.3±6.5	ELISA
Hou XX(2016)	2016	China	Asians	64	30	34/30	18/12	66.8±7.3	69.7±6.8	ELISA
Guo CF(2016)	2016	China	Asians	70	70	41/29	43/27	56.2±3.8	55.8±3.5	ELISA
Yan WR(2015)	2015	China	Asians	47	60	25/22	30/30	68.59±8.7	66.91±9.5	ELISA
Chen FF(2015)	2015	China	Asians	38	35	27/11	NR	60.5(42~6	NR	ELISA
Wei GY(2013)	2013	China	Asians	153	114	85/68	65/49	58.5 ± 12.	NR	ELISA
Li SS(2013)	2013	China	Asians	42	28	NR	14/14	NR	68.8±6.9	ELISA
Sarchielli P-a(2013)	2013	Italy	Caucasian	28	31	6/22	11/20	64 (53~70)	36 (28~50	Luminex x
Sarchielli P-b(2013)	2013	Italy	Caucasian	21	31	10/11	11/20	71(67~73)	36 (28~50	Luminex x
Jefferis BJ(2013)	2013	UK	Caucasian	300	590	191/109	372/218	71.3 ± 5.3	71.3 ± 5.3	ELISA
Sun J(2013)	2013	China	Asians	79	60	40/39	30/30	68.6(60~7	66.5(60~7	ELISA
Zhao Y(2012)	2012	China	Asians	83	32	58/25	20/12	61.35±13.	58.32±12.	ELISA
Wang YJ(2011)	2011	China	Asians	218	218	NR	NR	NR	NR	ELISA
Ormstad H(2011)	2011	Norway	Caucasian	45	40	27/18	20/20	67.7 ± 11.	59.1 ± 5.7	Luminex x
Stott DJ-a(2009)	2009	UK	Caucasian	445	532	NR	270/262	NR	75.9 ± 3.6	ELISA
Stott DJ-b(2009)	2009	UK	Caucasian	179	532	NR	270/262	NR	75.9 ± 3.6	ELISA
Bossu P(2009)	2009	Italy	Caucasian	30	25	14/16	12/13	66.4 ± 14.	68.6 ± 7.2	ELISA
Welsh P(2008)	2008	UK	Caucasian	472	1011	348/124	750/261	67 (66~67	66 (66~67	ELISA
Yuen CM(2007)	2007	China	Asians	217	20	136/81	12/8	66.3 ± 10.	67.1 ± 9.3	ELISA
Zaremba J(2003)	2003	Poland	Caucasian	23	15	6/17	4/11	72.2 ± 10.	70.1 ± 8.6	ELISA

Notes: M = male; F = female; NR = not reported; a: silent brain infarcts; b:lacunar stroke.

### Main results of the meta-analysis

**Figure 3:**
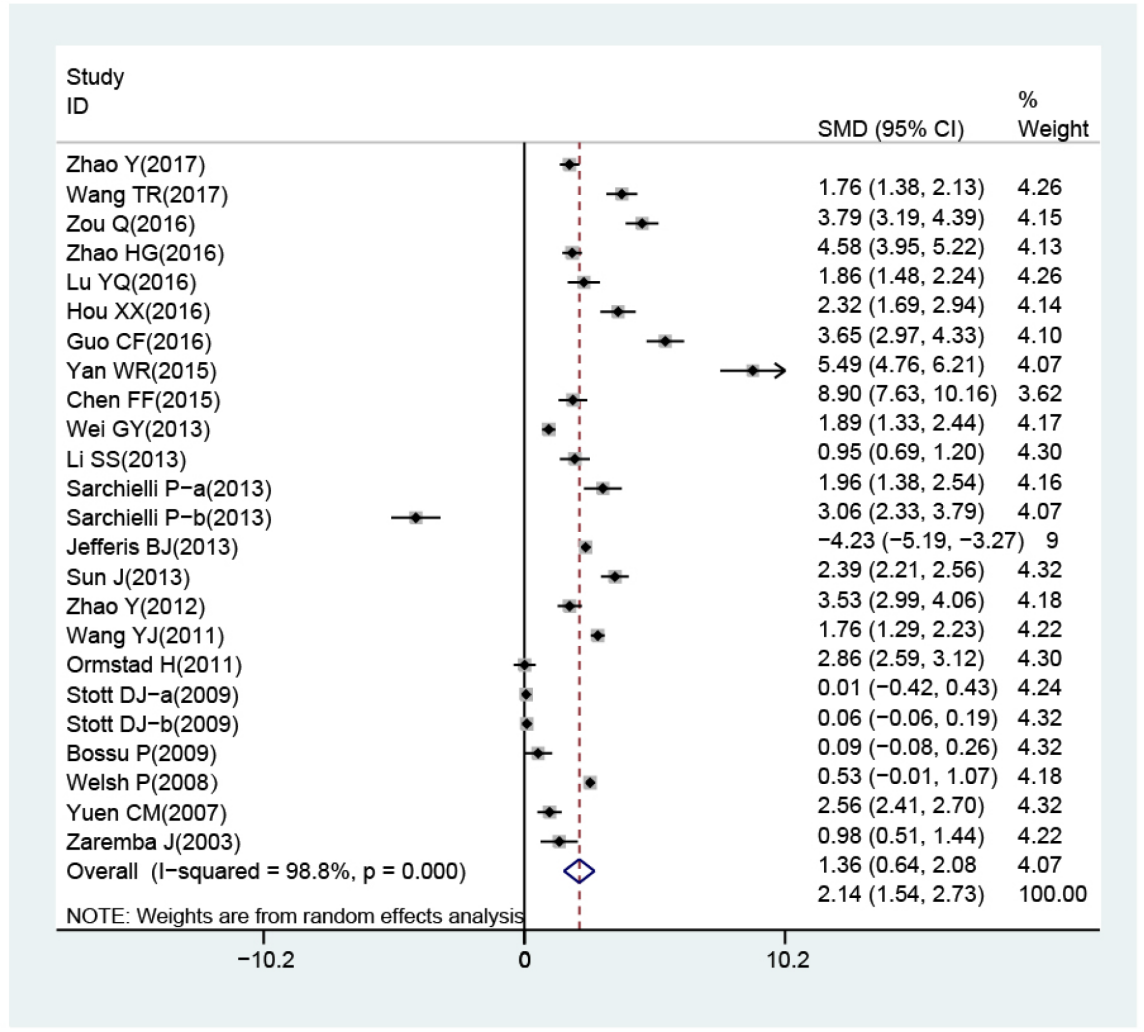
**Forest plots for the difference of interleukin-18 levels between stroke patients and healthy controls.**

The random-effects model for the heterogeneity test showed the association between IL-18 level and the risk of stroke (case vs. control: *I*^2^ = 98.8%, *p*< 0.001). IL-18 level was higher in patients than in controls (case vs. control: SMD = 2.14, 95% CI = 1.54 ~ 2.73, *p*< 0.001) (Figure 3).

**Figure 4:**
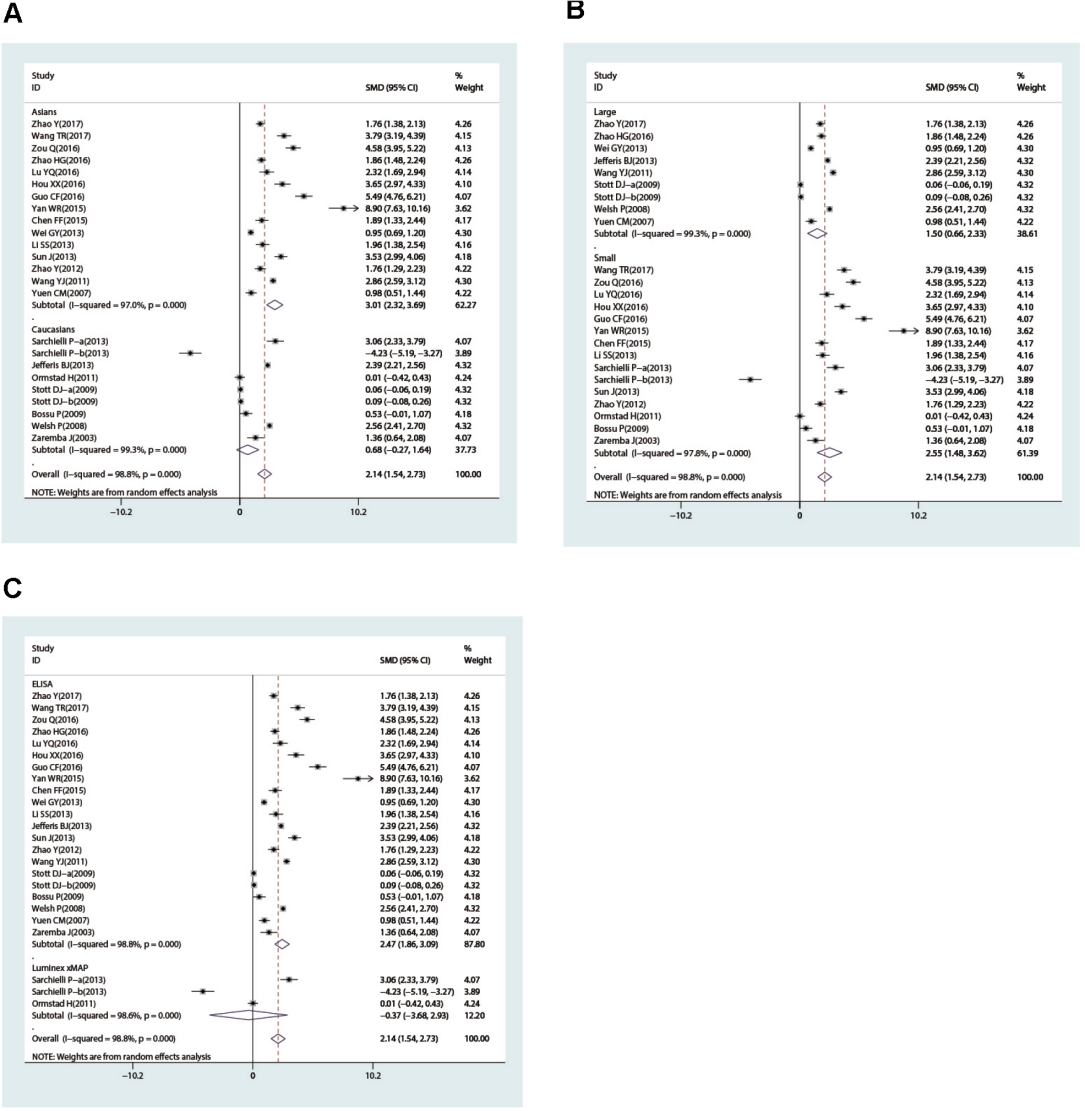
Subgroup analyses for the difference of interleukin-18 levels between stroke patients and healthy controls (**A**, race; **B**, sample size; **C**, detection method).

In the subgroup analysis stratified by country, we found that IL-18 level was higher in patients than in controls in China (SMD = 3.01, 95% CI = 2.32∼3.69, *p*< 0.001), Norway (SMD = 2.86, 95% CI = 2.25~3.46, *p*< 0.001), and Poland (SMD = 1.36, 95% CI = 0.64~2.08, *p*< 0.001) but not in Italy or the UK (all *p*> 0.05). The ethnicity-stratified subgroup analysis showed that increased IL-18 level was associated with stroke in the Caucasian (case vs. control: SMD = 0.72, 95% CI = 0.12~1.31, *p* = 0.018) and Asian subgroups (case vs. control: SMD = 1.60, 95% CI = 0.24~2.96, *p* = 0.021) (Figure 4A). Additionally, high IL-18 level was associated with susceptibility to stroke in the large-sample-size subgroup (SMD = 1.04, 95% CI = 0.40~1.68, *p* = 0.001) but not in the small sample size subgroup (*p*> 0.05) (Figure 4B). Furthermore, in the subgroup analysis stratified by measurement method, high IL-18 level was associated with susceptibility to stroke in the ELISA subgroup (SMD = 1.02, 95% CI = 0.46~1.58, *p*< 0.001) but not the Luminex xMAP subgroup (*p*> 0.05) (Figure 4C).

**Figure 5:**
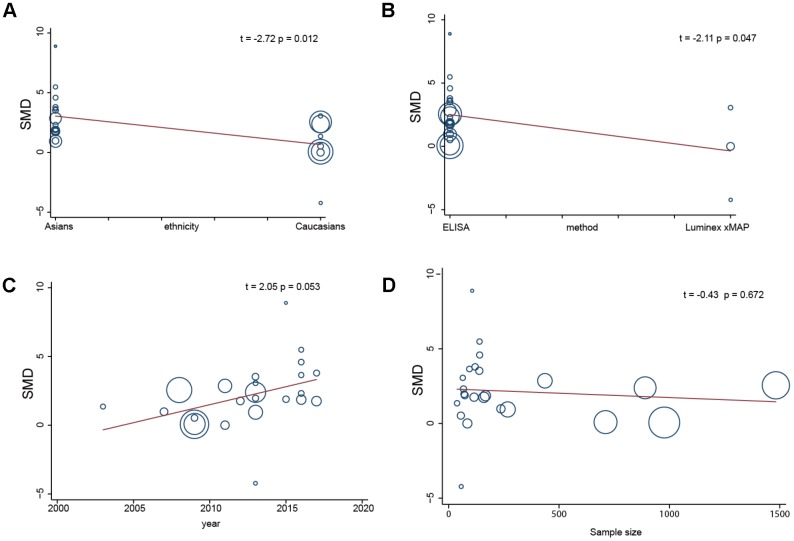
Single factor meta regression analysis (**A**, race; **B**, detection method; **C**, years; **D**, sample size).

To further identify the sources of heterogeneity, we performed a single-factor meta-analysis on race, age, sample size and method. We found that race and method may be the main sources of heterogeneity (race: *p* = 0.012; method: *p* = 0.047) (Figure 5A and 5B), while age and sample size may not be related to heterogeneity (age: *p* = 0.053; sample size: *p* = 0.672) (Figure 5C and 5D). Race, age, sample size and method were introduced into the regression model, and the SMD and *p* values indicated that the 4 compound variables were not associated with the heterogeneity among studies (race: *p* = 0.579; method: *p* = 0.285; age: *p* = 0.885; sample size: *p* = 0.302).

### Secondary findings of the meta-analysis

Analysis of the severity of disease demonstrated that IL-18 level in patients with mild, moderate and severe ischemic stroke was significantly higher than those in controls (mild: SMD = 1.74, 95% CI = 0.57 ~ 2.92, *p* = 0.004; moderate: SMD = 3.89, 95% CI = 1.72 ~ 6.06, *p*< 0.001; severe: SMD = 8.10, 95% CI = 4.40 ~ 11.80, *p*< 0.001) (Supplementary Figure 2). IL-18 level in patients with severe ischemic stroke was higher than those in patients with moderate ischemic stroke (SMD =3.33, 95% CI = 1.37 ~ 5.29, *p* = 0.001) (Supplementary Figure 3). IL-18 level in patients with moderate ischemic stroke was higher than those in patients with mild ischemic stroke (SMD =1.79, 95% CI = 0.90 ~ 2.68, *p*< 0.001) (Supplementary Figure 4). All of the aforementioned findings revealed that the levels of IL-18 increased as the severity of ischemic stroke increased.

### Sensitivity analysis and publication bias evaluation

**Figure 6:**
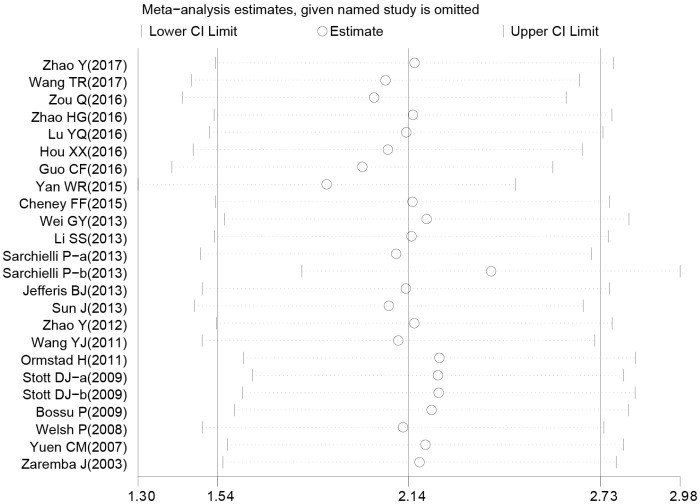
**Sensitivity analysis of the difference of interleukin-18 levels between stroke patients and healthy controls.**

**Figure 7:**
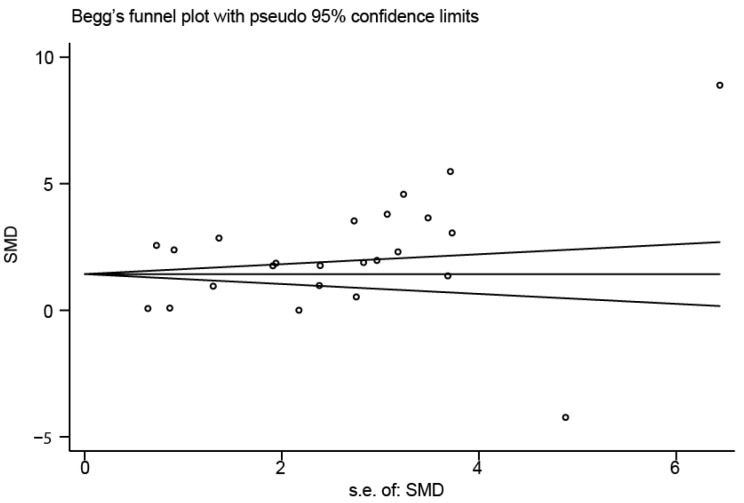
**Funnel plot of publication biases on the difference of interleukin-18 levels between stroke patients and healthy controls.**

Sensitivity analysis was used to detect whether any single included study could influence the results. This analysis showed that no single study had an outsize effect on the pooled SMDs (Figure 6), and the inverted funnel plot was symmetrical, indicating no obvious publication bias. Asymmetry tests of the funnel plot showed *p* = 0.189 by Begg’s rank correlation method and *p* = 0.098 by Egger’s linear regression method, further confirming that there was no obvious publication bias (Figure 7).

### DISCUSSION

Previous results regarding the association between IL-18 level and ischemic stroke have been inconsistent. In the present study, we conducted a cross-sectional case-control study in patients with first-episode ischemic stroke and followed it with a meta-analysis to validate our results. We found in the cross-sectional study that IL-18 level in stroke patients was higher than those in controls. Interestingly, IL-18 level was negatively correlated with NIHSS scores, indicating that IL-18 was positively correlated with illness severity. Our subsequent meta-analysis supported the results of our cross-sectional study. Our meta-analysis demonstrated significantly higher IL-18 level in patients than in controls. Additionally, IL-18 level was higher in patients with severe ischemic stroke than in patients with moderate ischemic stroke, and IL-18 level was higher in patients with moderate ischemic stroke than in patients with mild ischemic stroke. The results of our study have revealed a strong link between higher levels of IL-18 and the development and severity of ischemic stroke. Furthermore, the subgroup analysis and single-factor meta-analysis showed that ethnicity and detection methods may be the main sources of heterogeneity among previous studies.

IL-18 is synthesized by macrophage cells, blood mononuclear cells and neurons. It is described as an inducing factor of IFN-γ and a pro-inflammatory cytokine that results in the expression of pro-inflammatory cytokines, chemokines, and Fas ligand as well as the activity of some inflammatory cells, such as neutrophils, macrophages, and T and NK cells [14]. In addition to its role in acquired and innate immunity as a pleiotropic pro-inflammatory cytokine, IL-18 has been reported to be linked with increased carotid intima-media thickness and the propagation and instability of atherosclerotic plaques [16, 17]. Ischemic stroke, the most common type of stroke, can induce a robust inflammatory reaction, producing molecules that are important for inflammatory signaling, particularly cytokines that are expressed in immune cells, glial cells, and neurons; thus, upregulation of cytokines can be detected after stroke [31]. High levels of IL-18 have been found in unstable carotid plaques, leading to high IL-18 level after ischemic stroke [24]. Furthermore, aside from inducing IFN-γ, IL-18 can also induce expression of TNF-α and synthesis of IL-10, cytokines that can inhibit the inflammatory process, leading to the instability of atherosclerosis plaques and formation of thromboses, a main risk factor for stroke [17]. Furthermore, increased IL-18 level is associated with increased expression of other inflammatory and hemostatic markers (including IL-6, C-reactive protein, fibrinogen, viscosity, factor VIII and fibrin D-dimer) as well as increased blood pressure and lipid concentrations, which are the main cardiovascular risk factors [30]. We can conclude from the above analysis that high IL-18 level is closely linked to the development of stroke through their connection to many types of inflammatory cytokines, which can lead to changes in atherosclerosis plaques, thrombosis formation, hypertension and hyperlipidemia, which ultimately result in the development of stroke. Paola *et al.* also found that IL-18 level was significantly linked to the severity of stroke and the severity of alexithymia, indicating lesions of the right hemisphere [26].

Considering other related factors that could possibly affect the association between higher IL-18 level and the pathogenesis of stroke, a stratified analysis based on countries, ethnicity, sample size and protein measurement method was conducted. From the country-stratified and ethnicity-stratified analyses, we were able to conclude that the association was significant in both Asians and Caucasians; while there was no obvious correlation in Italy or the UK, there were strong associations in China, Norway and Poland, a fact that may be explained by differences in lifestyles between countries. In conclusion, our results were consistent with other studies demonstrating that high IL-18 level is strongly linked to ischemic stroke. These results suggest that IL-18 is a useful diagnostic and prognostic indicator for ischemic stroke.

Concerning the limitations of our study, our findings are constricted by the lack of longitudinal assessments of IL-18 level in larger stroke cohorts together with clinical findings, especially regarding the specific location of lesions. In this regard, data from larger sample sizes on the numbers and locations of lesions are therefore warranted for a comprehensive interpretation of our results. Second, we did not classify stroke subtypes to examine the IL-18 level among these different subgroups. The third limitation arises from the existence of heterogeneity, as the included measurement methods and ethnic backgrounds may not be comparable. Additionally, gender and age information was not detailed in the studies conducted by Wang YJ *et al*. and Stott DJ *et al*. Thus, certain publication conventions may restrict a broader analysis of research data.

## CONCLUSIONS

In conclusion, our present study provides evidence for the involvement of IL-18 in stroke. IL-18 level markedly increased in stroke and were positively related to the severity of stroke. IL-18 level might be considered a potential biomarker for stroke.

## MATERIALS AND METHODS

### Cross-sectional study

### Subjects

The present study was reviewed and approved by the Institutional Review Boards of Renji Hospital. Written informed consent was obtained from each participant or his or her guardian. Inpatients were recruited from December 2015 to December 2017 at Renji Hospital, Shanghai Jiao Tong University School of Medicine. All patients recruited in the present study satisfied the following inclusion criteria: (1) age ≥40 years, (2) no prior history of stroke or transient ischemic attack, (3) stroke duration equal or less than 72 hours, (4) fulfillment of the diagnostic criteria of ischemic stroke [45], and (5) positive MRI detection of stroke. Stroke is defined as a rapidly developing global or focal brain dysfunction lasting for more than 24 hours, unless interrupted due to surgery or death, without apparent nonvascular causes [45]. A total of 252 stroke patients were recruited with an age of 66.4 ± 10.5 years, 175 males and 77 females, with an average stroke duration of 32.01±4.54 hours.

Healthy subjects were recruited as controls. A total of 259 healthy controls (165 males and 94 females) with a mean age of 44.22 ± 12.50 years were recruited in the present study. There was a significant difference in age between patients and healthy controls (t=21.78, p<0.001) but no difference in gender (χ^2^=2.17, p=0.14).

Subjects with a history of recent infections, immune system diseases, malignancy, febrile disorders, or abnormal blood counts and/or C-reactive protein (CRP) were excluded.

### Serum IL-18 measurement

Whole blood samples were collected by forearm vein puncture from fasting participants during the hours of 9:00 am-11:00 am. Samples were prepared using standard protocols and centrifuged at 4000×*g* for 5 min. The supernatants were aliquoted into polypropylene tubes stored at −80°C. Serum IL-18 level was measured in duplicate by ELISA (R&D Systems, USA).

### NIHSS and mRS assessment

Two neurologists applied the NIH Stroke Scale (NIHSS) to assess the severity of the patients’ condition at the time of admission. Stroke severity was stratified according to NIHSS score as follows: mild (NIHSS<6), moderate (NIHSS 6-16), and severe (NIHSS>16) [46].

Researchers were trained simultaneously to perform the NIHSS, and the consistency and reliability of ratings were examined. The interobserver correlation coefficient for the NIHSS was 0.84.

mRS [47] assessment was employed at the time of discharge to assess the short-term outcome, which was the primary outcome measure for acute stroke trials. The scale ranges from 0 to 6, representing conditions from no symptoms to death.

### Meta-analysis design

### Search strategy

Potentially relevant studies were first identified through a comprehensive literature search without restrictions regarding study language, design or data collection. We searched the following bibliographic databases: PubMed (1966~2017), Embase (1974~2017), Science Citation Index (1945~2017), Cochrane Library (Oxford, UK, Issue 12, 2017), CINAHL (1982~2017), and Current Contents Index (1995~2017). Additionally, Chinese-language articles were identified using three databases: Weipu Journal (1989~2017), Chinese Journal Full Text (1980~2017), and Chinese Biomedical (1978~2017). We used a highly sensitive search strategy to search for the following subject headings with no restrictions on language: (“Stroke” or “Brain Infarction” or “Cerebral Infarction” or “Cerebral Ischemic Stroke” or “Cerebral Stroke” or “Ischemic Stroke”) and (“Interleukin-18” or “Interleukin-18 Receptors” or “IL-18” or “IL-18 Receptors” or “Receptors, Interleukin-18” or “Interferon-gamma-inducing Factor”). In addition, other relevant publications were identified by manually searching the reference lists of the selected studies.

### Inclusion and exclusion criteria of the publications

The following criteria were used for evaluating the retrieved studies: (1) the studies were conducted in humans and published in peer-reviewed journals; (2) the studies used a case-control design and investigated the IL-18 level in stroke; (3) stroke was identified by the diagnostic criteria established by the MONICA project and by brain imaging with MRI or CT [45]; (4) the studies had original or sufficient data on IL-18 level; (5) when studies providing overlapping data, the one with the most information was selected. The following categories were excluded: (1) studies that did not meet the inclusion criteria; (2) some types of publication including abstracts, letters, meta-analyses, reviews, and proceedings; (3) unpublished data; and (4) duplicate publications without extractable numerical data. The title and abstract of each article were assessed for relevance. Among the relevant articles, the full texts were carefully examined to decide their eligibility for inclusion.

### Study quality and data extraction

Two researchers independently reviewed and evaluated the methodological quality of the included results using the Newcastle-Ottawa Scale (NOS) criteria, and the scores given by the two researchers were compared to confirm their consistency [48]. Possible NOS scores range from 0 to 9, and three domains are scored: (1) subject selection: 0~4; (2) subject comparability: 0~2; (3) clinical outcome: 0~3. Studies were required to score ≥ 7 in order to be included.

Each reviewer evaluated the data independently according to the inclusion and exclusion criteria. A standardized data form was used to extract the following information: initials and surname of the first author, name of the journal, publication or submission year, publication language, numbers of cases and controls, study design, country, race of subjects, demography of subjects, method of IL-18 measurement, IL-18 level in the cases and controls and confirmation of diagnosis. Inconsistencies in the inclusion of studies were resolved by group discussion or consultation with the third reviewer.

### Statistical analysis

All data in the cross-sectional study were analyzed in SPSS and Stata 13.1 (StataCorp, College Station, TX). A one-sample nonparametric test was used to test the distribution of data. As serum IL-18 level was not normally distributed, we used a log transformation to normalize the data distribution. For continuous variables and categorical variables, we used Student’s t-test and the chi-squared (χ^2^) test, respectively. Regarding the difference in age between patients and healthy controls, analysis of covariance (ANCOVA) was applied to compare the IL-18 level between the two groups, with IL-18 level as the dependent variable, diagnosis (patients or controls) as the independent variable, and age as the covariate. Partial correlation and multivariate regression were used to examine the association of IL-18 level with NIHSS scores, mRS scores, and infarction volume, adjusting for age, gender, BMI, smoking and duration of stroke. ROC analysis was used to calculate the cutoffs for different levels of illness severity.

Standardized mean difference (SMD) was applied to calculate the effect size of the meta-analysis. The *Z* test was applied to calculate the 95% confidence interval (95% CI) for the summary SMD. Additionally, the heterogeneity among the included studies was examined by Cochran’s *Q*-test and the *I^2^* test [27]. If the *Q*-test showed *P*< 0.05 or the *I^2^* value was > 50%, it indicated that the included studies were in the highest heterogeneity class, and meta-regression analyses with a random-effects model were conducted to examine the sources of heterogeneity; otherwise, SMDs were calculated using a fixed-effects model [49, 50]. The possible sources of heterogeneity were evaluated by single-factor and multifactor meta-regression analysis, and multiple comparisons were performed using a Monte Carlo simulation [51, 52]. Furthermore, in order to assess the effect of a single study on the total estimate, potential publication bias should be examined using a funnel plot and Egger’s linear regression with visual inspection of the funnel plot [53]. All tests were two-sided. *P*< 0.05 was taken to indicate statistical significance.

### Ethics approval

The present study was reviewed and approved by the Institutional Review Boards of Renji Hospital. Written informed consent was obtained from each participant or their guardians.

## Supplementary Material

Supplementary Figures
