# Supplementary material for: Increased interleukin-18 level contributes to the development and severity of ischemic stroke
Source: Aging (Albany NY). 2019 Sep 16;11(18):7457–72. doi: 10.18632/aging.102253 (PMC6781996; doi:10.18632/aging.102253)
Supplement: Supplementary Figures [file aging-11-102253-s001.pdf]

SUPPLEMENTARY FIGURES

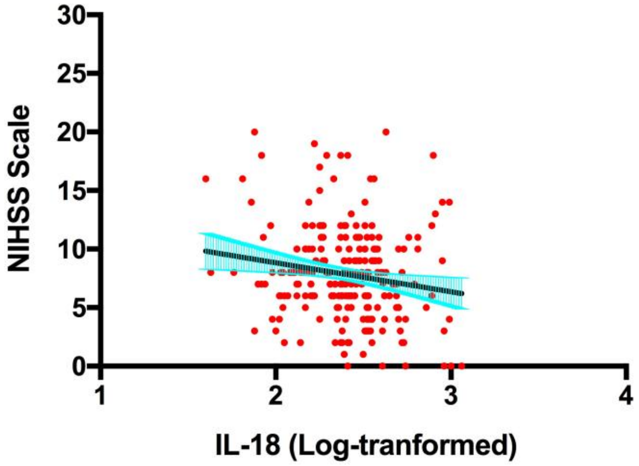

Supplementary Figure 1. The correlation between IL-18 levels and NIHSS scale in patients.

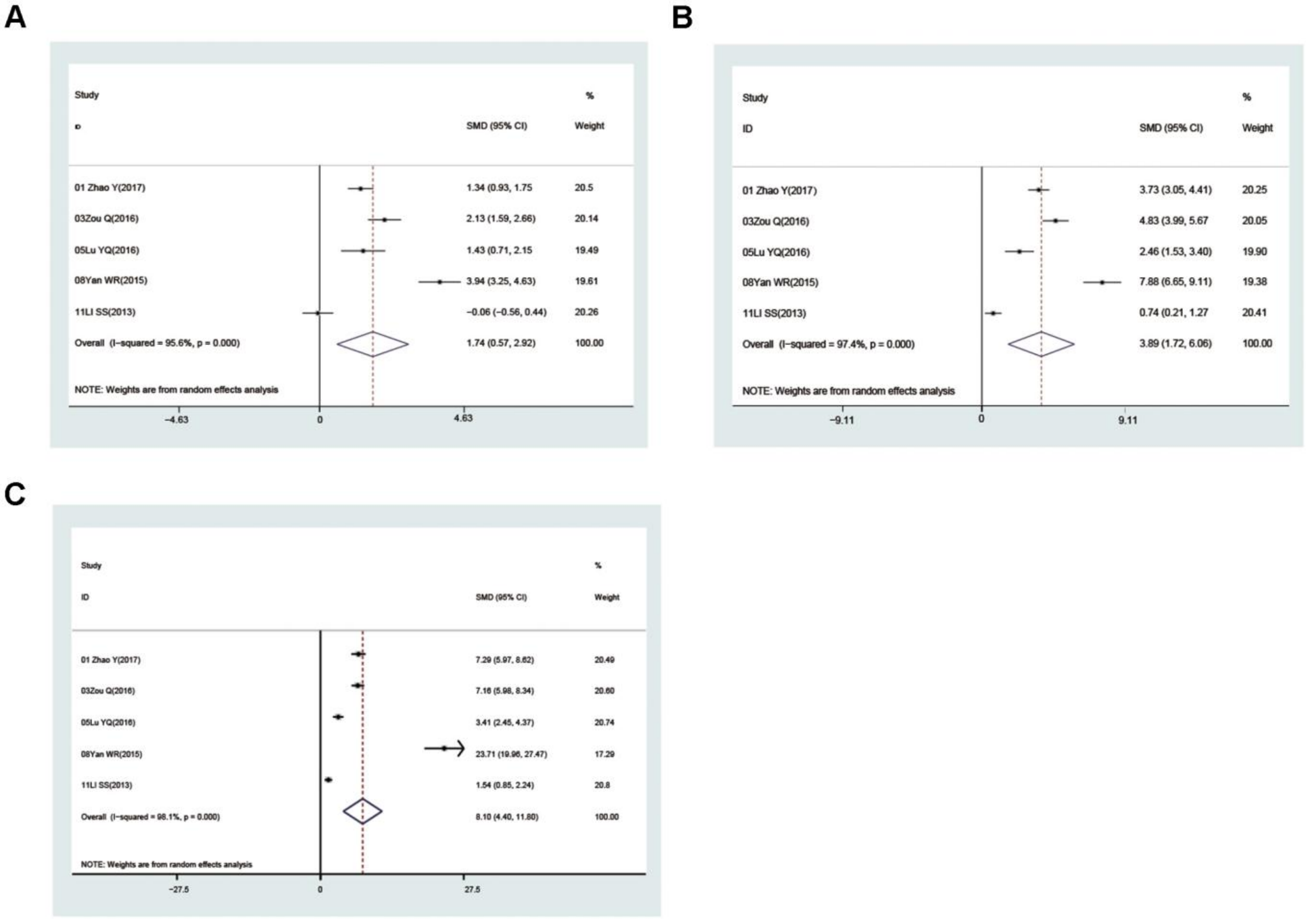

Supplementary Figure 2. Comparison of IL-18 levels in patients with different severity of cerebral infarction and normal healthy individuals (**A**: mild, **B**: moderate, **C**: severe).

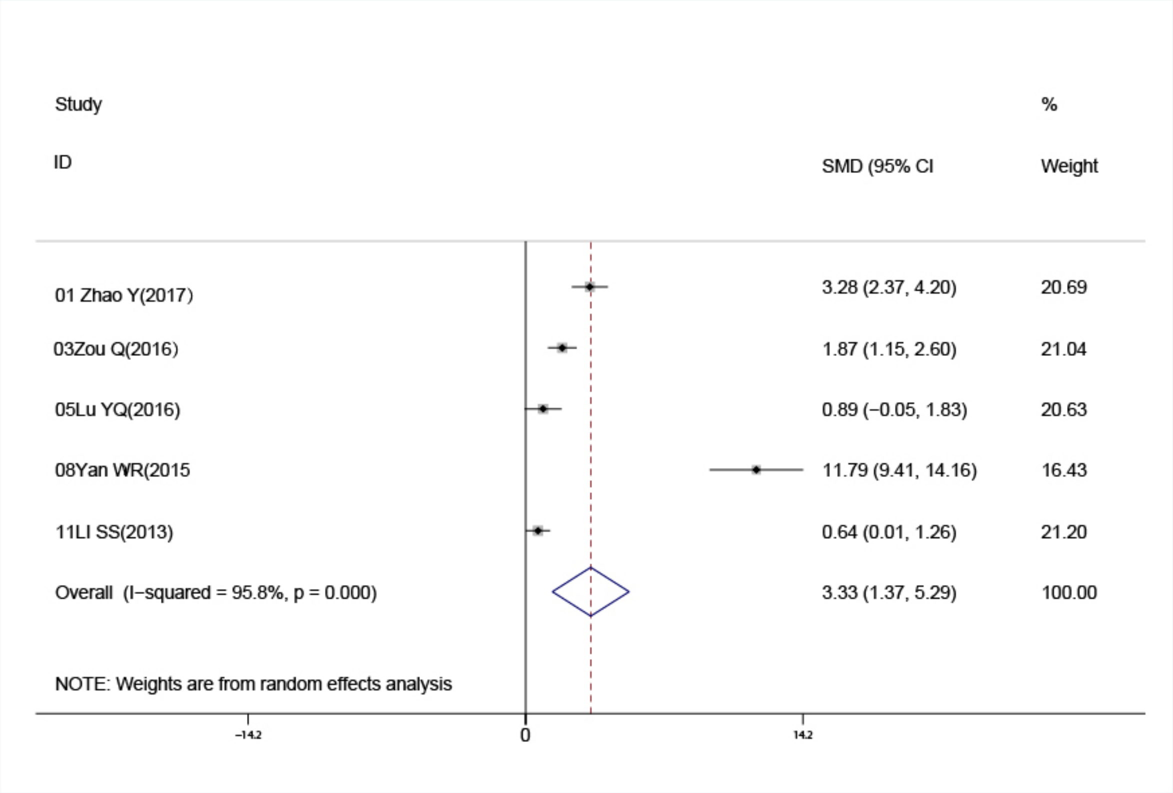

Supplementary Figure 3. Comparison of IL-18 levels between severe cerebral infarction patients and moderate cerebral infarction patients.

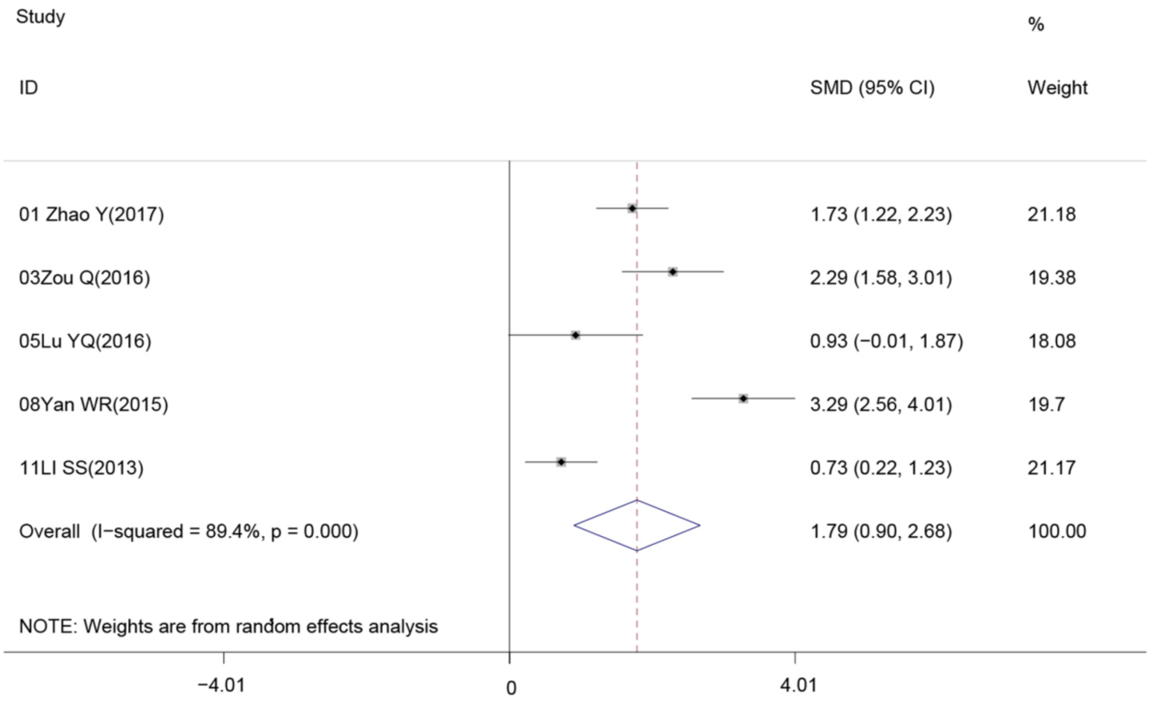

Supplementary Figure 4. Comparison of IL-18 levels between severe cerebral infarction patients and mild cerebral infarction patient.
